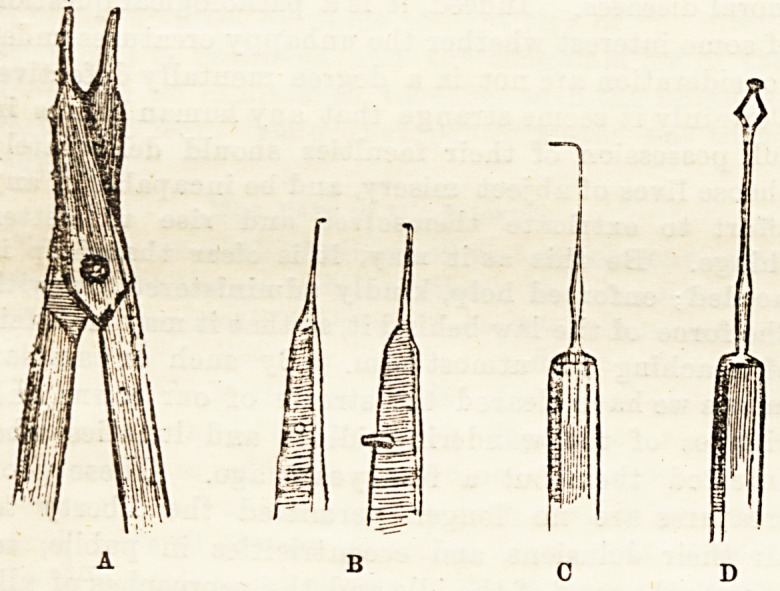# On Modern Progress in Ophthalmic Medicine and Surgery

**Published:** 1896-01-18

**Authors:** Robert Brudenell Carter

**Affiliations:** Consulting Ophthalmic Surgeon to St. George's Hospital.


### ON MODERN PROGRESS IN OPHTHALMIC MEDICINE AND SURGERY.

By ROBERT BRUDENELL CARTER, F.R.C.S., Consulting Ophthalmic Surgeon to St. George's Hospital.

SQUINT (*Continued from page* 245).

IT has been reserved for American surgeons to introduce and to bring to perfection methods of operating for squint which render it possible to determine the effect produced with very great precision, and which have set aside many of the risks of failure enumerated in the last paper. Great attention has lately been given in America to conditions of imperfect equilibrium of the eye muscles, of which I shall have to speak more fully hereafter, and means have been sought of strengthening any muscle or of weakening its antagonist in such a degree that correct equilibrium should be restored. The operations devised for the fulfilment of this purpose, and for which we are mainly indebted to Dr. Stevens, of New York, have afforded great facilities for dealing also with declared forms of squint, and the results of their employment have thrown light upon the previously obscure causes of the occasional failure of a well-devised and well-executed operation of the old kind. Just as a body free to move, and drawn by equal forces acting at right angles to each other, will follow the diagonal between them, so the position of an eye,

A B C D

whether correct or deviating, is not governed by the action of any single muscle, but by the sum of the forces exerted by all the individuals of the group by which it is controlled. There are cases in which an internal squint is not originally due to over-action of the internus muscle, or to weakness of its antagonist, but to excess or defect of the superior or inferior rectus, the tendency to a faulty position in an upward or downward direction being only overcome by the rest of the group at the cost of a squint. A tendency of the two eyes to rest in slightly different horizontal planes is excessively disturbing to vision, and is therefore unconsciously opposed; while an original inward squint is often so large that the image of the deviating eye is received upon a peripheral part of the retina, and may be so ignored by the consciousness as not to disturb vision at all. In the former case, a tenotomy of the internus will not be sufficient to bring about a correct position, and may even aggravate the defect which it is intended to cure.

What may be called the American operation for squint is done with the instruments shown in the figure, and the most important of these are the scissors, A, and the forceps, B. The former are made with the last half-inch of the blades very fine, smooth, and rounded externally, very sharp, and cutting accurately to the points within. The forceps also terminate in very fine blades, with "mouse-tooth" points. In order to perform tenotomy, the eye is prepared by cocaine, or the patient is anæsthetised, and the surgeon pinches up a minute fold of conjunctiva just over the middle of the insertion of the muscle. This fold is then snipped through with the scissors, so as to make an opening barely large enough to allow the closed blades to enter it. The opening is dilated by the outer sides of the scissor blades, and the forceps are then passed through it, and made to seize the middle of the tendon, which is lifted from the sclera, and cut through in a similar manner. The very fine hook, C, shown in the figure is then passed through the hole in the tendon, followed by one blade of the scissors, while the other blade passes between the tendon and the conjunctiva, and the opening is enlarged first towards one border of the tendon and then towards the other, the scissors being kept as close as possible to the sclera. If only a very small effect is desired, the edges of the tendon may be left uncut, but, generally speaking, the whole of it should be divided. When the operation is completed, the wound in the conjunctiva is scarcely more than a puncture, and, except for the severance of the tendon from its absolute scleral attachment, none of the ocular tissues have been disturbed or divided. No dressing or after treatment is required.

A tenotomy so strictly limited as this may be performed, when necessary, upon both eyes at once, without any fear of producing ultimate divergence in a squint of ordinary magnitude; but the best results have lately been attained by limiting the operation to the squinting eye, and by advancement of the antagonist in addition to tenotomy of the contracted muscle. Thus, in an ordinary convergent squint, limited to the left eye, the operator would divide the tendon of the left internus, and would advance that of the left externus. The first steps of the operation for advancement resemble those for tenotomy, but the tendon is carefully separated from its attachments by the scissors, and by the instrument shown at D in the figure, which has a probe point and small lateral cutting edges. This may be passed through the hole in the tendon, and used to liberate it from the sclera, as well as from the conjunctiva, until the tendon can be drawn into and through the conjunctival wound, in which position it may be firmly held by a barbed hook. The hole in the tendon is then enlarged so as to cut out a piece of sufficient size, leaving the borders intact, and the opening in the tendon is then drawn together and closed by a single thread of very fine silk, which may be cut and removed after three or four days. The advantage of this proceeding is that the tendon is not completely severed from the sclera, so that it cannot retract, and failure to unite if such an event were to occur, would leave the position no worse than it was before.

A still better method of performing advancement has lately been devised by Dr. Black. Two needles are threaded with fine silk, and the two ends of each thread are knotted together. One needle is passed through the conjunctiva and superficial layer of the sclera, near the corneal margin, and in a direction towards it, just above the margin of the tendon, and the needle is passed through the loop formed by the knotted ends of its thread and is drawn tight. The other is passed in the same way, but below the margin of the tendon. The tendon is then exposed by two horizontal scissor cuts, parallel to its margins, and is freed from its attachments. The two needles are passed through it, from within outwards, as far back as may be required, as much as necessary of the portion nearest the insertion is cut away, the threads are drawn tight, and tied together over a little plate of aluminium, which is perforated for the passage of the needles. The silk may be cut and removed in four days, and no trace of the operation will be visible after the lapse of three or four weeks.

## Figures and Tables

**A B C D f1:**